# Relationship between knee isokinetic muscle strength and countermovement jump performance in elite male football players

**DOI:** 10.1186/s13102-026-01722-9

**Published:** 2026-05-01

**Authors:** Denis Čaušević, Cristina Ioana Alexe, Ensar Abazović, Elena Adelina Panaet, Nedim Čović, Dan Iulian Alexe

**Affiliations:** 1 Faculty of Sport and Physical Education, University of Sarajevo, Bosnia and Herzegovina, Sarajevo, 71000 Bosnia and Herzegovina; 2 Department of Physical Education and Sports Performance, “Vasile Alecsandri” University of Bacau, Bacău, 600115 Romania; 3 Department of Physical and Occupational Therapy, “Vasile Alecsandri” University of Bacau, Bacău, 600115 Romania

**Keywords:** Soccer, Isokinetic strength, Countermovement jump, Angular velocity, Inter-limb asymmetry

## Abstract

**Background:**

Countermovement jump (CMJ) performance is widely used to assess explosive lower-limb function in football players. Although knee isokinetic strength is frequently measured in elite sport environments, the extent to which it relates to CMJ performance remains unclear, particularly when CMJ is performed with free arm movement. Therefore, the aim of this study was to examine the relationship between knee isokinetic muscle strength characteristics and CMJ performance in elite male football players.

**Methods:**

Twenty-four elite male football players (age 23.83 ± 5.98 years) participated in this cross-sectional study. CMJ height was assessed using an optical measurement system (Optojump Next). Concentric knee extensor and flexor peak torque was measured using an isokinetic dynamometer at angular velocities of 60°/s and 180°/s and expressed as peak torque/body weight% (PT/BW,%). Pearson correlation and linear regression analyses were used to examine associations between isokinetic strength variables and CMJ performance. Bilateral differences, hamstring-to-quadriceps (H/Q) ratios, and inter-limb asymmetries were also analyzed.

**Results:**

Significant positive correlations were observed between CMJ height and knee extensor peak torque expressed as PT/BW (%) at both angular velocities. Stronger relationships were found at 180°/s (*r* = 0.558–0.642, *p* ≤ 0.005) compared with 60°/s (*r* = 0.483–0.500, *p* < 0.05). Regression analyses showed that knee extensor strength at 180°/s explained up to 41.2% of the variance in CMJ height. Hamstring strength demonstrated weaker and less consistent associations with CMJ performance, while H/Q ratios and inter-limb asymmetries were not significantly related to jump height.

**Conclusion:**

Quadriceps isokinetic strength expressed as PT/BW (%) was significantly associated with CMJ performance in elite male football players, with stronger relationships observed at higher angular velocity. These findings suggest that knee extensor strength assessed at higher angular velocity is meaningfully associated with explosive lower-limb performance and may provide useful complementary information within routine neuromuscular monitoring in professional football.

## Introduction

Vertical jump performance is a critical component of explosive lower-limb function in football and is closely associated with essential match-related actions such as sprinting, jumping for aerial duels, and executing rapid changes of direction [1, 2]. Among various jump assessments, the countermovement jump (CMJ) is widely used due to its simple execution, high reliability, and sensitivity in evaluating neuromuscular performance [3]. Consequently, CMJ height is frequently adopted as a global indicator of explosive lower-limb capacity, particularly in elite football players [4].

The mechanical determinants of CMJ performance are inherently complex, involving coordinated interactions across multiple joints and muscle groups throughout the kinetic chain. Within this framework, the knee extensors play a pivotal role in vertical force production, especially during the propulsion phase of the jump [5]. As a result, laboratory-based assessments of knee muscle strength—most notably isokinetic dynamometry—have been extensively employed to quantify maximal force-generating capacity and to examine its relationship with explosive performance outcomes [5–7]. Isokinetic testing enables precise control of angular velocity and provides reliable measures of peak torque, inter-limb asymmetry, and hamstring-to-quadriceps (H/Q) ratios, which are commonly applied in performance diagnostics and injury risk screening in football [8, 9].

Despite the widespread application of isokinetic testing, previous studies investigating the relationship between knee isokinetic strength and CMJ performance have reported inconsistent findings. While some research has demonstrated moderate to strong associations between quadriceps strength and CMJ or squat jump height, other studies have reported weak or non-significant relationships [2, 10, 11]. One plausible explanation for these discrepancies relates to differences in testing velocity. Lower angular velocities (e.g., 60°/s) primarily reflect maximal strength capacity [12], whereas higher velocities (e.g., 180°/s) may better represent the neuromuscular demands of explosive movements, where force must be generated rapidly within limited time constraints [13, 14].

Methodological aspects related to CMJ execution further complicate interpretation of existing evidence [15]. Earlier studies frequently assessed CMJ performance using restricted arm movement (hands-on-hips), which reduces jump height and alters coordination strategies [16]. In contrast, CMJ performed with a free arm swing more closely reflects sport-specific jumping tasks and is therefore considered a more ecologically valid assessment of explosive performance [2, 17, 18]. However, relatively few studies have examined whether isolated laboratory-based strength measures retain explanatory value for CMJ performance when assessed under such less constrained and more natural conditions. In addition to absolute strength measures, the monitoring of inter-limb asymmetries and H/Q ratios has gained prominence in elite football as indicators of neuromuscular balance and potential injury risk. Nevertheless, the relationship between these variables and CMJ performance remains unclear, particularly in well-trained professional players who are exposed to continuous monitoring and targeted conditioning programs.

Therefore, the purpose of the present study was to examine the relationship between knee isokinetic muscle strength characteristics and CMJ performance in elite male football players within a professional club monitoring context. Specifically, this study aimed to: (1) determine the associations between concentric knee extensor and flexor strength at two angular velocities (60°/s and 180°/s) and CMJ height; (2) evaluate the predictive value of these strength variables for CMJ performance; and (3) explore bilateral differences, inter-limb asymmetry, and H/Q ratios in relation to CMJ height. It was hypothesized that knee extensor strength, particularly at higher angular velocity, would demonstrate stronger associations with CMJ performance than knee flexor strength, while asymmetry and H/Q ratios would contribute limited explanatory value.

## Materials and methods

### Study design

A cross-sectional correlational study design was employed to examine the relationship between knee isokinetic muscle strength characteristics and countermovement jump (CMJ) performance in elite male football players. The primary objective was to determine the extent to which concentric knee extensor and flexor strength, as well as inter-limb strength asymmetries, are associated with vertical jump height as an indicator of lower-limb explosive performance. Isokinetic muscle strength of the knee joint was assessed under standardized laboratory conditions at two angular velocities (60°/s and 180°/s), reflecting distinct strength and speed-related neuromuscular demands. Peak torque values were normalized to body weight and expressed as peak torque/body weight% (PT/BW, %) to enable inter-individual comparison, and bilateral strength asymmetry indices were calculated to provide additional information on potential functional imbalances.

Associations between isokinetic strength variables and CMJ height were explored using correlational and regression-based analytical approaches. All measurements were conducted within a single testing period under controlled conditions. Participants were instructed to maintain normal hydration status and to refrain from strenuous physical activity for at least 24 h, as well as from food and caffeine intake for at least 3 h prior to testing.

### Participants

A total of 24 elite male football players (age = 23.83 ± 5.98 years) voluntarily participated in this study. All participants were actively competing at the professional level at the time of testing. The data analyzed in the present study were collected as part of an ongoing player monitoring program conducted in collaboration with FK Sarajevo; however, only cross-sectional data from a single testing session were included in the analysis. All measurements were performed during the preseason period as part of the club’s routine performance monitoring program.

Inclusion criteria required participants to be free from lower-limb musculoskeletal injuries for at least six months prior to testing and to have no history of knee surgery. Players reporting acute pain, recent injury, or any medical condition that could affect maximal performance were excluded from the study. Body composition was assessed using bioelectrical impedance analysis (InBody BSM 370 and InBody 720, Biospace Co., Seoul, Republic of Korea). These data were used for descriptive purposes only.

An a priori power analysis was conducted using G*Power software (version 3.1.9.7; Heinrich Heine University Düsseldorf, Germany) [19], to estimate the required sample size. The analysis was performed with the following parameters: α error probability = 0.05, statistical power (1–β) = 0.80, and an assumed coefficient of determination (effect size) of 0.50. Based on these assumptions, the minimum required sample size was estimated to be *n* = 10 participants. The final sample size in the present study (*n* = 24) exceeded this requirement and is comparable to previous cross-sectional studies conducted in professional football players using intra-team analyses and correlation approaches [20, 21].

The study was conducted as part of a club-approved monitoring program. All procedures were performed in agreement with the club’s medical and performance staff. Players were informed about the purpose and procedures of the assessments through the club, and participation was voluntary. Written informed consent was obtained in accordance with club regulations. The study was conducted in accordance with the Declaration of Helsinki and approved by the relevant institutional ethics committee (Approval No: 01–56/26).

### Testing procedure

All testing procedures were conducted during the preseason period at the club’s performance testing facility. Testing was performed on an indoor synthetic sports surface under controlled environmental conditions (temperature ~ 21–23 °C). All assessments were supervised and conducted by experienced sport scientists and strength and conditioning staff familiar with the testing protocols. Participants were tested in small groups of 3–4 players to ensure standardized procedures and adequate recovery between trials. The testing sequence was standardized for all participants. First, anthropometric and body composition measurements were collected. This was followed by a standardized warm-up consisting of 10 min of low-intensity cycling and dynamic stretching exercises targeting the lower-limb musculature. After the warm-up, participants performed the countermovement jump (CMJ) test. Following completion of the jump assessments and an additional rest period of approximately 5 min, knee isokinetic strength testing was conducted using an isokinetic dynamometer.

#### Countermovement jump (CMJ) test

Countermovement jump (CMJ) performance was assessed using an optical measurement system (Optojump Next, Microgate, Bolzano, Italy)(ICC = 0.98) [22]. The CMJ was performed with free arm movement to allow a natural jumping pattern and to better reflect sport-specific explosive performance in football players. Participants started from an upright standing position and executed a rapid downward countermovement followed immediately by a maximal vertical jump [23]. They were instructed to jump as high as possible while freely using their arms during the movement. The depth of the countermovement was self-selected in order to avoid alterations in individual jumping coordination.

Prior to testing, participants performed two submaximal familiarization jumps followed by one maximal practice attempt to ensure proper execution of the movement. Subsequently, each participant performed three maximal CMJ trials, with a standardized rest period of 60 s between attempts. The highest jump height (cm) achieved across the three trials was used for subsequent statistical analyses.

#### Knee isokinetic muscle strength assessment

Knee isokinetic muscle strength was assessed using an isokinetic dynamometer (Biodex System, Biodex Medical Systems, USA). Testing was performed for the knee extensors and flexors of both limbs under concentric muscle action conditions. The dominant limb was defined as the preferred kicking limb, determined by asking each player which leg they would use to kick a ball with maximal force and accuracy. Prior to isokinetic testing, participants completed a standardized warm-up protocol consisting of 10 min of low-intensity cycling followed by dynamic stretching exercises targeting the lower-limb musculature [24]. Subsequently, participants performed several submaximal familiarization repetitions on the dynamometer to become accustomed to the testing movement and angular velocities.

Participants were tested in a seated position with the hip joint flexed at approximately 85°. The trunk, pelvis, and thigh of the tested limb were stabilized using straps to minimize compensatory movements. The axis of rotation of the dynamometer was aligned with the lateral femoral epicondyle of the knee joint, and gravity correction was applied prior to testing [25]. The limb testing order was fixed and standardized across all participants, with the dominant limb tested first, followed by the non-dominant limb. Adequate rest was provided between limbs and testing velocities to minimize fatigue effects.

The range of motion was set from 90° of knee flexion to near full extension (10°). Isokinetic testing was conducted at two angular velocities: 60°/s and 180°/s. At each angular velocity, participants performed five maximal voluntary repetitions, with strong verbal encouragement provided to ensure maximal effort [26]. Adequate rest periods were allowed between testing conditions to minimize fatigue. Peak torque (PT) values were recorded for both knee extensors and flexors of each limb. For the present analyses, peak torque values normalized to body weight were used and expressed as peak torque/body weight% (PT/BW, %) in order to account for differences in body size between participants and allow more meaningful comparisons of relative muscle strength. Furthermore, the hamstring-to-quadriceps strength ratio (H/Q ratio) was calculated as the ratio between peak torque of the knee flexors and knee extensors. Bilateral strength asymmetry was calculated using the following formula [27]:

$$\mathrm{Asymmetry}\;\left({\%}\right)=\left(\left(\mathrm{stronger}\;\mathrm{limb}\;-\;\mathrm{weaker}\;\mathrm{limb}\right)\;/\;\mathrm{stronger}\;\mathrm{limb}\right)\times100$$

These variables were included to provide additional insight into inter-limb strength differences and muscle balance characteristics relevant to injury risk and performance in football players.

### Statistical analyses

Statistical analyses were performed using SPSS Statistics software (IBM Corp., Armonk, NY, USA). Descriptive statistics are presented as mean ± standard deviation (SD), with 95% confidence intervals (CI) calculated where appropriate. The normality of data distribution was assessed using the Shapiro–Wilk test. Relationships between CMJ height and isokinetic knee strength variables were examined using Pearson’s product–moment correlation coefficients. The magnitude of correlation coefficients was interpreted as trivial (< 0.10), small (0.10–0.30), moderate (0.31–0.50), large (0.51–0.70), very large (0.71–0.90), and nearly perfect (> 0.90) [28]. Simple linear regression analyses were conducted to determine the extent to which selected isokinetic strength variables predicted CMJ performance. Paired-samples t-tests were used to examine bilateral differences between the dominant and non-dominant limbs for isokinetic knee strength variables and hamstring-to-quadriceps (H/Q) ratios at both angular velocities (60°/s and 180°/s). Because multiple related correlation and regression analyses were performed, Bonferroni-adjusted significance thresholds were additionally considered as a sensitivity check. Exact p-values are reported, and the findings were interpreted cautiously due to the exploratory nature of the analyses and the relatively small sample size. The level of statistical significance was set at *p* < 0.05 for all analyses.

## Results

Descriptive characteristics of the participants are presented in Table 1. The mean age of the players was 23.83 ± 5.98 years, with a mean body height of 184.22 ± 7.01 cm and a mean body mass of 79.76 ± 6.43 kg. Mean body fat percentage was 10.89 ± 3.29%, while the mean body mass index was 23.49 ± 1.10 kg/m². The mean CMJ height was 47.38 ± 6.16 cm.

**Table 1:** Anthropometric, body composition, and performance characteristics of elite male football players (*n* = 24)

Variables	Mean	SD	95% CI	
			Lower	Upper
Age	23.83	5.98	21.31	26.36
Height (cm)	184.22	7.01	181.26	187.17
Body weight (kg)	79.76	6.43	77.05	82.49
PBF (%)	10.89	3.29	9.50	12.28
BMI (kg/m^2^)	23.49	1.10	23.02	23.96
CMJ height (cm)	47.38	6.16	44.78	49.98

Isokinetic peak torque values of the knee extensors and flexors expressed as peak torque/body weight% (PT/BW, %) at angular velocities of 60°/s and 180°/s are shown in Table 2. At both angular velocities, significantly higher peak torque values were observed in the dominant limb compared with the non-dominant limb for both knee extensors and flexors (*p* < 0.05). Hamstring-to-quadriceps (H/Q) ratios for both angular velocities are presented in Table 2. No significant bilateral differences were observed for the H/Q ratio at either 60°/s or 180°/s (*p* > 0.05). Inter-limb asymmetry values for knee extensors and flexors at both testing velocities are also presented in Table 2. Mean asymmetry values ranged from approximately 5% to 8% across muscle groups and angular velocities.

**Table 2:** Isokinetic knee strength, hamstring-to-quadriceps ratio, and inter-limb asymmetry at 60°/s and 180°/s expressed as peak torque/body weight% (PT/BW, %)

Variables	Non-dominant leg Mean ± SD (95%CI)	Dominant leg Mean ± SD (95%CI)	T - test	
			*p*	Cohen’s d
Knee extensor PT/BW at 60°/s (%)	315.37 ± 39.04 (298.88–331.85)	325.05 ± 32.73 (311.23–338.87)	0.016	0.53
Knee flexor PT/BW at 60°/s (%)	178.12 ± 23.77 (168.08–188.16)	189.11 ± 25.62 (178.30–199.93)	0.002	0.73
Knee extensor PT/BW at 180°/s (%)	215.45 ± 24.07 (205.29–225.61)	222.03 ± 21.89 (212.78–231.27)	0.043	0.44
Knee flexor PT/BW at 180°/s (%)	143.87 ± 18.18 (136.20–151.55)	148.65 ± 19.28 (140.51–156.79)	0.023	0.50
H/Q ratio at 60°/s	57.10 ± 7.21 (54.06–60.14)	58.00 ± 5.98 (55.47–60.52)	0.700	0.08
H/Q ratio at 180°/s	66.55 ± 6.60 (63.76–69.34)	67.10 ± 7.30 (64.02–70.19	0.441	0.16

Inter-limb asymmetry (%)	Mean ± SD (95% CI)			
Knee extensor asymmetry at 60°/s	5.21 ± 3.77 (3.62–6.80)			
Knee flexor asymmetry at 60°/s	7.76 ± 5.40 (5.48–10.04)			
Knee extensor asymmetry at 180°/s	5.55 ± 4.68 (3.58–7.53)			
Knee flexor asymmetry at 180°/s	5.53 ± 4.02 (3.84–7.23)			

Correlations between isokinetic knee extensor strength expressed as PT/BW (%) and CMJ height are presented in Table 3. Significant positive correlations were observed between CMJ height and knee extensor PT/BW (%) for both limbs at both angular velocities. At 60°/s, CMJ height showed moderate correlations with knee extensor strength in the dominant (*r* = 0.500, *p* = 0.013) and non-dominant limbs (*r* = 0.483, *p* = 0.017). At 180°/s, higher correlations were observed for both the dominant (*r* = 0.558, *p* = 0.005) and non-dominant limbs (*r* = 0.642, *p* < 0.001).

**Table 3:** Correlations between quadriceps isokinetic strength expressed as PT/BW (%) and CMJ height

Variables	*r*	*p*
Peak torque of knee extensors at 60°/s (Nm/kg) – Dominant leg	0.500	0.013*
Peak torque of knee extensors at 60°/s (Nm/kg) – Non-dominant leg	0.483	0.017*
Peak torque of knee extensors at 180°/s (Nm/kg) – Dominant leg	0.558	0.005**
Peak torque of knee extensors at 180°/s (Nm/kg) – Non-dominant leg	0.642	< 0.001**

* *p* < 0.05; ** *p* < 0.01

**Fig. 1:**
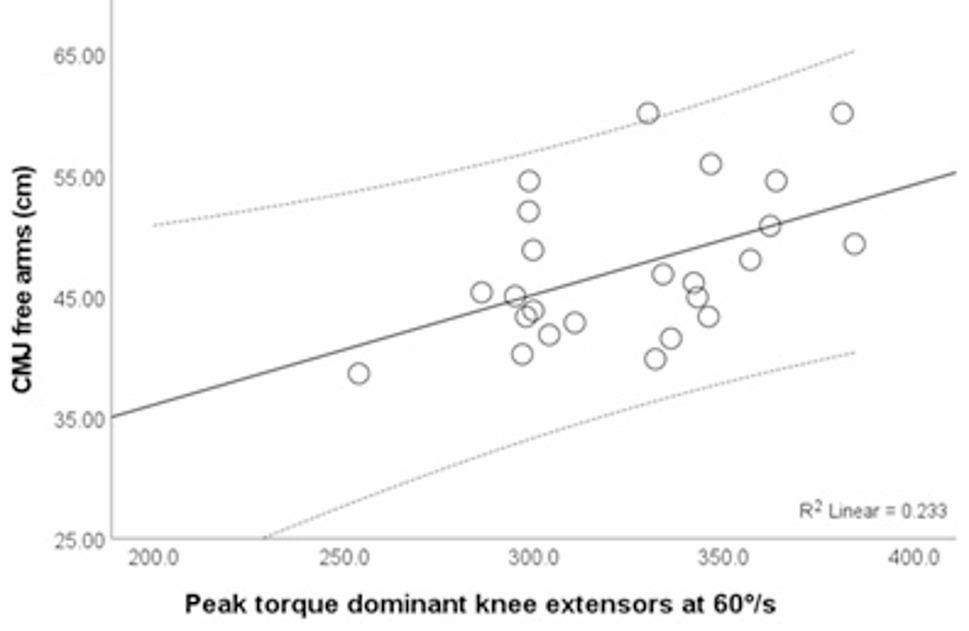
Relationship between countermovement jump (CMJ) height (free arms) and peak torque of the dominant knee extensors at 60°/s expressed as PT/BW (%). The solid line represents the linear regression, while dashed lines indicate the 95% confidence intervals (R² = 0.233)

**Fig. 2:**
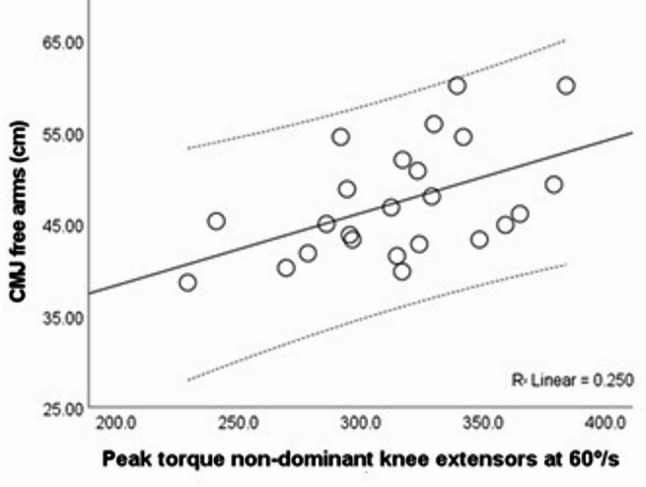
Relationship between countermovement jump (CMJ) height (free arms) and peak torque of the non-dominant knee extensors at 60°/s expressed as PT/BW (%). The solid line represents the linear regression, while dashed lines indicate the 95% confidence intervals (R² = 0.250)

**Fig. 3:**
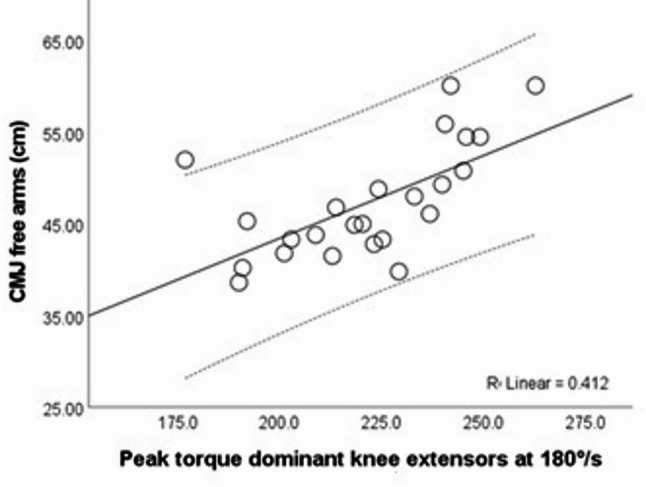
Relationship between countermovement jump (CMJ) height (free arms) and peak torque of the dominant knee extensors at 180°/s expressed as PT/BW (%). The solid line represents the linear regression, while dashed lines indicate the 95% confidence intervals (R² = 0.412)

**Fig. 4:**
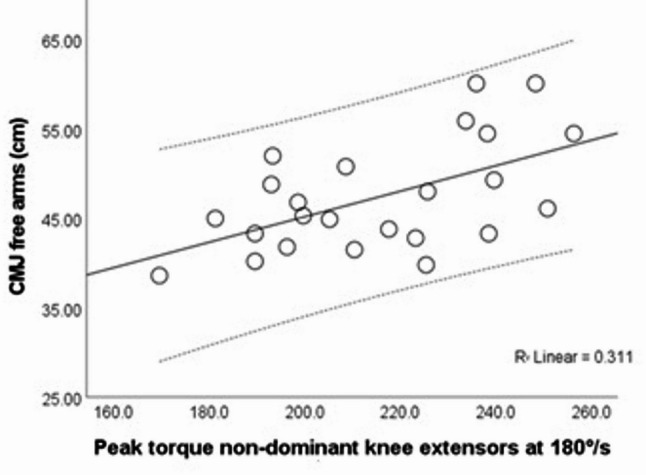
Relationship between countermovement jump (CMJ) height (free arms) and peak torque of the non-dominant knee extensors at 180°/s expressed as PT/BW (%). The solid line represents the linear regression, while dashed lines indicate the 95% confidence intervals (R² = 0.311)

**Fig. 5:**
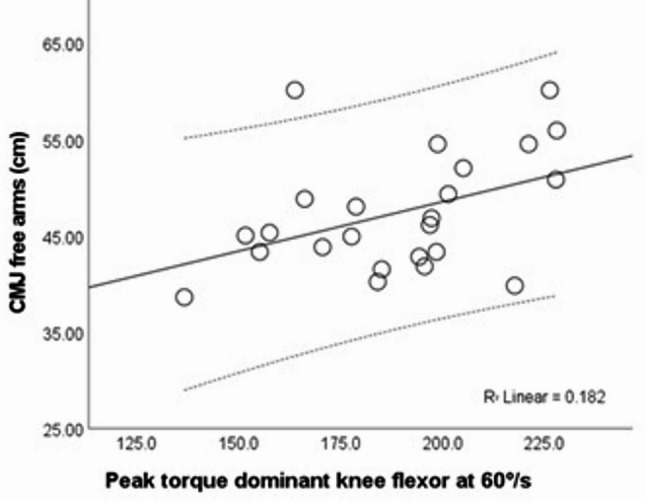
Relationship between countermovement jump (CMJ) height (free arms) and peak torque of the dominant knee flexors at 60°/s expressed as PT/BW (%). The solid line represents the linear regression, while dashed lines indicate the 95% confidence intervals (R² = 0.182)

**Fig. 6:**
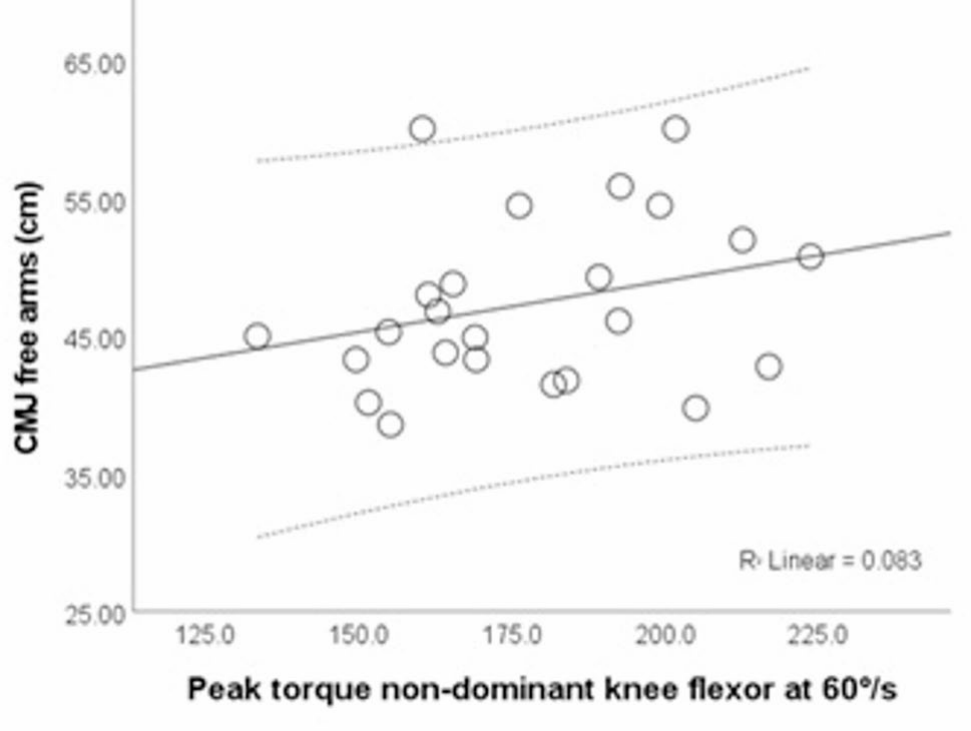
Relationship between countermovement jump (CMJ) height (free arms) and peak torque of the non-dominant knee flexors at 60°/s expressed as PT/BW (%). The solid line represents the linear regression, while dashed lines indicate the 95% confidence intervals (R² = 0.083)

**Fig. 7:**
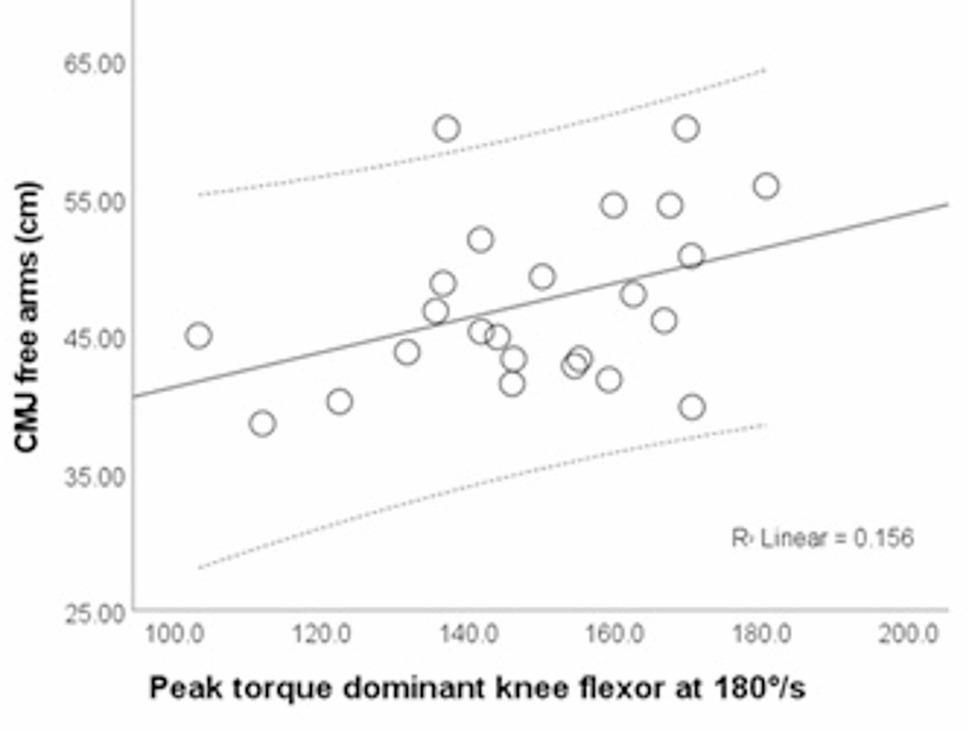
Relationship between countermovement jump (CMJ) height (free arms) and peak torque of the dominant knee flexors at 180°/s expressed as PT/BW (%). The solid line represents the linear regression, while dashed lines indicate the 95% confidence intervals (R² = 0.156)

**Fig. 8:**
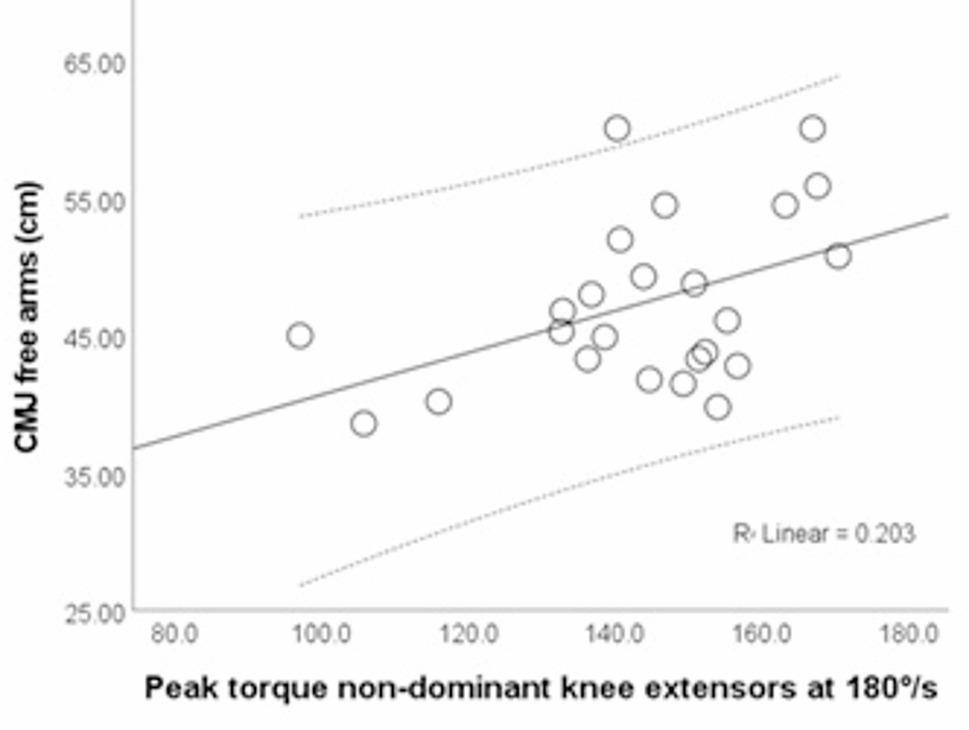
Relationship between countermovement jump (CMJ) height (free arms) and peak torque of the non-dominant knee flexors at 180°/s expressed as PT/BW (%). The solid line represents the linear regression, while dashed lines indicate the 95% confidence intervals (R² = 0.203)

At 60°/s, knee extensor PT/BW (%) was a significant predictor of CMJ height for both limbs. The dominant limb explained 25.0% of the variance in CMJ height (R² = 0.250, F = 7.346, *p* = 0.013), while the non-dominant limb explained 23.3% of the variance (R² = 0.233, F = 6.680, *p* = 0.017). Knee flexor PT/BW (%) at 60°/s showed a weaker predictive relationship, with the dominant limb explaining 18.2% of the variance (R² = 0.182, F = 4.882, *p* = 0.038), whereas the non-dominant limb did not significantly predict CMJ height (R² = 0.083, F = 1.984, *p* = 0.173). At 180°/s, knee extensor PT/BW (%) demonstrated stronger predictive capacity. The dominant limb explained 31.1% of the variance in CMJ height (R² = 0.311, F = 9.931, *p* = 0.005), while the non-dominant limb explained 41.2% of the variance (R² = 0.412, F = 15.434, *p* < 0.001). For knee flexor PT/BW (%) at 180°/s, the dominant limb did not significantly predict CMJ height (R² = 0.156, F = 4.062, *p* = 0.056), whereas the non-dominant limb explained 20.3% of the variance in CMJ height (R² = 0.203, F = 5.608, *p* = 0.027) (Figs. 1, 2, 3, 4, 5, 6, 7 and 8). Associations between H/Q ratios, inter-limb asymmetry variables, and CMJ height were also examined. None of the H/Q ratio variables or inter-limb asymmetry indices showed significant correlations with CMJ height (all *p* > 0.05), indicating that these composite measures were not meaningfully associated with vertical jump performance in the present cohort.

## Discussion

The present study examined the relationship between knee isokinetic muscle strength and countermovement jump (CMJ) performance in elite male football players, with additional emphasis on bilateral differences, hamstring-to-quadriceps (H/Q) ratios, and inter-limb asymmetry. The main findings show that quadriceps peak torque expressed as PT/BW (%) was consistently associated with CMJ height at both 60°/s and 180°/s, with stronger relationships and greater explained variance observed at 180°/s. In contrast, hamstring strength showed weaker and less consistent associations, while H/Q ratios did not differ between limbs and were not related to CMJ performance.

A key contribution of the present study lies in the combined interpretation of correlation and regression analyses across two angular velocities within a professional club monitoring context. While previous studies have often reported associations between isokinetic knee extensor strength and vertical jump performance, the magnitude and functional relevance of these relationships appear to depend strongly on testing velocity and performance context [29, 30]. The stronger associations observed at 180°/s in the present study support the concept that moderate-to-high angular velocities may better reflect the neuromuscular demands of explosive movements, where force must be generated rapidly within constrained time windows [31, 32].

Recent biomechanical evidence suggests that free-arm CMJ places greater emphasis on coordinated force transmission across the kinetic chain rather than isolated joint torque production [33]. Within this context, the finding that quadriceps strength at 180°/s explained over 40% of CMJ variance in the dominant limb is particularly noteworthy, as it indicates that isokinetic knee extensor strength retains strong explanatory value even when CMJ is assessed using a less constrained, free-arm jumping protocol. Although the present study did not directly compare free-arm and hands-on-hips CMJ protocols, the use of a free-arm CMJ provides an ecologically relevant context for interpreting the association between isokinetic knee extensor strength and jump performance [34, 35].

The weaker and less consistent associations observed for hamstring strength are in line with recent literature indicating that knee flexors play a secondary role in vertical jump propulsion, contributing primarily to joint stabilization, energy transfer, and coordination rather than direct vertical impulse generation [32, 36, 37]. While hamstring strength has been associated with sprinting performance and hamstring injury risk in football [38, 39], its direct association with CMJ height appears weaker, particularly when jump performance is assessed with free arm movement. This may explain why hamstring peak torque showed lower predictive value in the regression models despite significant bilateral differences.

Another relevant finding is the absence of significant bilateral differences in H/Q ratios at both angular velocities. Contemporary research increasingly emphasizes that H/Q ratios should be interpreted primarily as indicators of neuromuscular balance and injury risk rather than performance determinants [40, 41]. The present results support this perspective, as symmetrical H/Q ratios were observed despite clear dominance-related differences in absolute strength. This suggests that elite football players may develop bilateral balance in antagonist muscle function even when preferential loading of the dominant limb occurs during sport-specific actions.

Inter-limb asymmetry values for both extensors and flexors were relatively low, ranging between approximately 5% and 8%. Recent systematic reviews have highlighted that asymmetry thresholds commonly used in applied practice (e.g., 10–15%) are highly task- and population-dependent and should not be interpreted as universal cut-off values [42, 43]. The low asymmetry values observed in the present cohort likely reflect the continuous monitoring and conditioning strategies implemented at the professional level and may partly explain why asymmetry measures were not strongly associated with CMJ performance. This further reinforces the notion that absolute and relative strength capacities may be more relevant for explosive performance than small inter-limb differences in well-trained athletes [44].

A further novel aspect of this study is that all data were derived from an ongoing professional player monitoring program, rather than from a one-off laboratory-based testing protocol. This context strengthens the ecological validity of the findings and supports the practical integration of isokinetic testing within routine performance diagnostics. Recent position statements advocate for combining laboratory-based strength measures with field-based performance tests to better inform decision-making in elite football environments [8, 45, 46]. The present findings align with this approach by suggesting that isokinetic quadriceps strength—particularly at 180°/s—may provide complementary information related to CMJ performance within a real-world monitoring framework [40].

Several limitations should be acknowledged. The cross-sectional design precludes causal inference, and the observed associations may reflect shared training adaptations rather than direct mechanistic relationships. Additionally, only concentric isokinetic strength at two angular velocities was assessed; inclusion of eccentric strength, rate-of-torque development, or higher testing velocities may further clarify the neuromuscular factors associated with explosive performance. Future longitudinal studies tracking seasonal changes in isokinetic strength and CMJ performance may provide deeper insight into the sensitivity of these measures to training adaptations and fatigue-related fluctuations.

## Conclusion

In elite male football players, quadriceps isokinetic strength expressed as PT/BW (%) was significantly associated with countermovement jump (CMJ) performance, with stronger associations observed at the higher angular velocity (180°/s). This pattern suggests that assessments of knee extensor strength at higher contraction velocities more closely reflect the neuromuscular demands underlying explosive lower-limb performance in football. In contrast, hamstring strength demonstrated weaker and less consistent associations with CMJ height, indicating that it may represent a less robust predictor of CMJ performance in this cohort. Ratios of hamstring-to-quadriceps strength and inter-limb asymmetries did not show meaningful relationships with CMJ performance, suggesting limited utility of these composite metrics for predicting CMJ height in elite players within the tested velocity range. Overall, the relative force-producing capacity of the knee extensors appears to be a relevant neuromuscular correlate of CMJ performance in elite football players. These findings suggest that high-velocity knee extensor strength assessments may be considered as part of routine neuromuscular monitoring and performance diagnostics in professional football. However, caution is warranted when generalizing these findings beyond trained male players or beyond the specific velocity conditions tested.

## Data Availability

The datasets used and/or analysed during the current study are available from the corresponding author on reasonable request.
